# Long-Term Sheep Implantation of WIMAGINE^®^, a Wireless 64-Channel Electrocorticogram Recorder

**DOI:** 10.3389/fnins.2019.00847

**Published:** 2019-08-21

**Authors:** F. Sauter-Starace, D. Ratel, C. Cretallaz, M. Foerster, A. Lambert, C. Gaude, T. Costecalde, S. Bonnet, G. Charvet, T. Aksenova, C. Mestais, Alim-Louis Benabid, N. Torres-Martinez

**Affiliations:** ^1^Univ. Grenoble Alpes, CEA, Leti, CLINATEC, Grenoble, France; ^2^Univ. Grenoble Alpes, CEA, Leti, DTBS, Grenoble, France

**Keywords:** long-term implantation, wireless communications, brain–computer interface, electrocorticogram (ECoG), signal quality, local tolerance, sheep

## Abstract

This article deals with the long-term preclinical validation of WIMAGINE^®^ (Wireless Implantable Multi-channel Acquisition system for Generic Interface with Neurons), a 64-channel wireless implantable recorder that measures the electrical activity at the cortical surface (electrocorticography, ECoG). The WIMAGINE^®^ implant was designed for chronic wireless neuronal signal acquisition, to be used e.g., as an intracranial Brain–Computer Interface (BCI) for severely motor-impaired patients. Due to the size and shape of WIMAGINE^®^, sheep appeared to be the best animal model on which to carry out long-term *in vivo* validation. The devices were implanted in two sheep for a follow-up period of 10 months, including idle state cortical recordings and Somato-Sensory Evoked Potential (SSEP) sessions. ECoG and SSEP demonstrated relatively stable behavior during the 10-month observation period. Information recorded from the SensoriMotor Cortex (SMC) showed an SSEP phase reversal, indicating the cortical site of the sensorimotor activity was retained after 10 months of contact. Based on weekly recordings of raw ECoG signals, the effective bandwidth was in the range of 230 Hz for both animals and remarkably stable over time, meaning preservation of the high frequency bands valuable for decoding of the brain activity using BCIs. The power spectral density (in dB/Hz), on a log scale, was of the order of 2.2, –4.5 and –18 for the frequency bands (10–40), (40–100), and (100–200) Hz, respectively. The outcome of this preclinical work is the first long-term *in vivo* validation of the WIMAGINE^®^ implant, highlighting its ability to record the brain electrical activity through the dura mater and to send wireless digitized data to the external base station. Apart from local adhesion of the dura to the skull, the neurosurgeon did not face any difficulty in the implantation of the WIMAGINE^®^ device and post-mortem analysis of the brain revealed no side effect related to the implantation. We also report on the reliability of the system; including the implantable device, the antennas module and the external base station.

## Introduction

Brain–Computer Interface (BCI) encompass various types of system, which have the common function of establishing a direct communication link between the brain and an external device. The large majority of these systems are non-invasive using a wearable cap to record brain activity from the scalp using either wet or dry Electroencephalography (EEG) electrodes. These systems have become very popular and range from single electrode systems (NeuroSky MindWave; Katona et al., 2016), to high-density EEG [up to 256 contacts (He and Sohrabpour, 2016)]. Among the BCI community, a large number of researchers are struggling to meet the needs of biofeedback-based applications and/or of clinical research, using non-invasive EEG recording systems with centimeter pitched contacts (Schwartz et al., 2006). These contacts record noisy signals generated by large cortical surfaces. In contrast, microelectrode-based systems tend to record single neurons or multi-unit activity. These systems became popular due to the first chronically implanted patients in the Braingate^®^ project (Hochberg et al., 2006), using UTAH arrays (Maynard et al., 1997) and the Cereport^®^ connector now retailed by BlackRock (Salt Lake City, UT, United States). However, due to mechanical mismatch between silicon and brain tissues, and volume displacement of the tissue following silicon needle introduction, glial encapsulation of the probe and neurodegeneration are likely (Schwartz et al., 2006). Accordingly, the number of usable contacts decreases dramatically with time (Rousche and Normann, 1998). Presenting a trade-off between invasiveness and signal quality, surface electrodes placed above (epidural) and below (subdural) the dura mater [electrocorticography (ECoG)] were reported to provide promising performances in BCI (Chao et al., 2010; Eliseyev and Aksenova, 2013; Wang et al., 2013). More recently in the framework of clinical trials, ECoG devices long-term assessment or set-up were reported, respectively for epileptic seizure detection (Cook et al., 2013; Sillay et al., 2013), closed-loop DBS (Swann et al., 2017) or BCI for a locked-in syndrome patient (Vansteensel et al., 2016).

Our team decided to explore the potential of epidural ECoG for chronic medical applications (e.g., motor BCI) using implanted electrodes at the surface of the dura mater to reduce glial reactions produced by penetrating microelectrodes, and to mitigate the lack of resolution of scalp electrodes (Schwartz et al., 2006). For the sake of patient safety and comfort, the implantable BCI recording device should be without wires. Consequently, we designed the wireless ECoG recording implant WIMAGINE^®^ using two antennas, one for the remote power supply at 13.56 MHz and the other for data communication in the Medical Implant Communication Service (MICS) band (402–405) MHz. Thanks to an overmolding of silicone rubber, the titanium housing looks like a cylinder of 50 mm in diameter, whose thickness varies between 7.5 and 12 mm at the center of the pseudo-spherical top surface, whereas the flat bottom surface is covered by 64 contacts for epidural ECoG plus 5 references. The WIMAGINE^®^ implant was developed as the starting point of a BCI platform which includes data processing, a Virtual Reality avatar and finally complex effectors such as a four-limb exoskeleton. The implant’s detailed description is given in a previous article (Mestais et al., 2015), including biocompatibility data resulting from the implantation of a semi-scaled and passive device over a 6-month period in a non-human primate model.

This article presents the ultimate step toward a clinical trial, the evaluation of a set of WIMAGINE^®^ implants for long-term functional assessment, and determination of the performance and stability of the WIMAGINE^®^ implant with a remote power supply and wireless data communication during chronic implantation in a large animal model. Experiments were carried out for more than 10 months, in freely moving animals to ensure stable functioning and to evaluate the signal quality.

To assess both the signal and the influence of surgical strategy on signal resolution, several long-term studies of epidural/subdural (Sillay et al., 2013; Gierthmuehlen et al., 2014; Ryapolova-Webb et al., 2014; Schendel et al., 2014; Degenhart et al., 2016; Kohler et al., 2017; Swann et al., 2017; Nurse et al., 2018), and even endovascular electrodes were carried out on large animals (Oxley et al., 2016). These studies lasted at least 4 months and could continue for up to 2 years. Considering the shape and size of the implant, we chose sheep for long-term implantation and monitoring, inspired by a previous 4-month sheep implantation experiment (Gierthmuehlen et al., 2014). Skull size in the adult sheep allows easy surgical implantation of WIMAGINE^®^ on the dura mater above sensorimotor areas. Moreover, these animals are easy to handle and are used to men, which allowed us to perform weekly recordings without anesthesia.

The quality of the signal recorded with a WIMAGINE^®^ implant and its evolution over time were investigated and compared to published clinical ECoG based studies (Sillay et al., 2013; Oxley et al., 2016; Swann et al., 2017; Nurse et al., 2018). Using the same experimental platform as that developed for the clinical trial, we recorded weekly raw ECoG signals and additional Somatosensory Evoked Potential sessions (SSEPs) every other month.

## Materials and Methods

### Wireless ECoG Recorder

**FIGURE 1:**
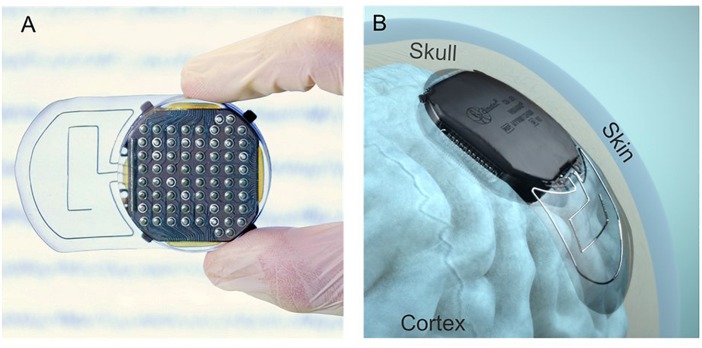
**(A)** View of the cortical electrode array. **(B)** Lateral implantation of a WIMAGINE^®^ implant on an anatomical model.

The WIMAGINE^®^ implant (Figure 1), consists of an array of 64 biocompatible epidural electrodes (Platinum iridium 90/10, 2.3 mm in diameter, pitches of 4 and 4.5 mm on the lateral and antero-posterior directions, respectively) fixed under a titanium housing including electronic boards, and two antennas for wireless transmission of data and a remote power supply. For this purpose, our team designed and handled the implant manufacture according to ISO 13485, as well as qualification tests according to the European directive 2007/47/EC and ISO standards (risk analysis ISO 14971, ISO14708-1 for electrical and mechanical safety of implantable devices, NF EN 60601-1 for electrical safety and electromagnetic compatibility of the external unit). A complete description of the WIMAGINE^®^ implant is given in Mestais et al. (2015).

The design of the WIMAGINE^®^ implant is adapted to a craniotomy with a trephine (50 mm in diameter) and the upper surface of the implant has a spherical shape (90 mm in curvature) that matches most of the patient’s skull. The implant is designed to replace the bone of the craniotomy. Four little titanium wings were added to prevent any mechanical damage to the brain in the case of mechanical pressure or shocks to the implant through the skin.

### Antenna Adaptation to Sheep

**FIGURE 2:**
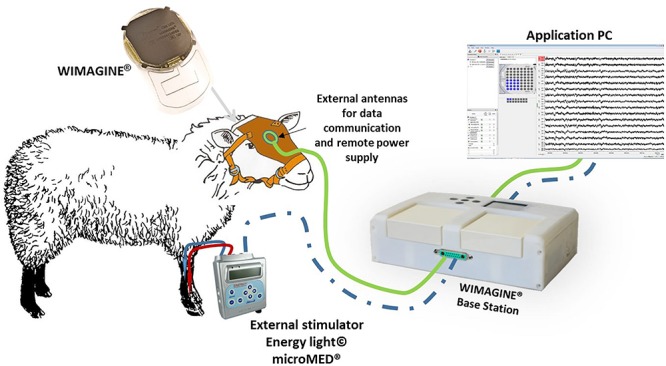
Experimental set-up for ovine experiments. The connection from the stimulator to the base station in dashed blue since it was only used to trig the stimulations to the recordings during the SSEP sessions.

In Figure 2, we sketched the experimental set-up of the ovine campaign. This consisted of a base station enabling communication between the implant and a laptop running the data recording software (WISCI) developed for the WIMAGINE^®^ platform: a telemetric antenna (402–405 MHz) and a remote power antenna (13.56 MHz) both included in a leather pocket attached to the sheep’s front to allow the recording awake animals in an idle state.

### Animals

Chronic implantation experiments were conducted on two female sheep (*Ovis Aries*) (Charollais breed, 80–90 kg, 5 and 8 years old). All experiments were carried out following the recommendations of the European Community Council Directives of 1986 (86/609/EEC), the National Institutes of Health Guide for the Care and Use of Laboratory Animals. The Ethics Committee COMETH of Lyon, France approved the experimental protocol which was registered to the national committee under reference number 1504_V2.

Animals were housed and kept together in an air-conditioned room under stable conditions of temperature (20 ± 2°C), humidity (50%), light (12 h light/dark cycle) and food/water was available *ad libitum*.

Sheep were observed daily and were clinically evaluated and weighed once a week by veterinary personnel.

### Implantation (Surgical Procedure)

After premedication (Diazepam 4.5 ml and Morphine 0.9 ml), anesthesia was induced with intravenously applied Propofol (4 mg/kg, 1/4 dose) and maintained using vaporized isoflurane (2%) in oxygen. Following endotracheal intubation, animals were maintained in volume-controlled ventilators at a respiratory rate of 12–14 breaths per minute. Fluid requirements were substituted by Ringer’s solution (Baxter, Deer Field, IL, United States) infused at 10 mg/kg. ECG, rectal body temperature and oxygen saturation were all monitored and the sheep were kept in a prone position during the procedure. A local anesthetic (2% Lidocaine) was injected prior to skin incision.

**FIGURE 3:**
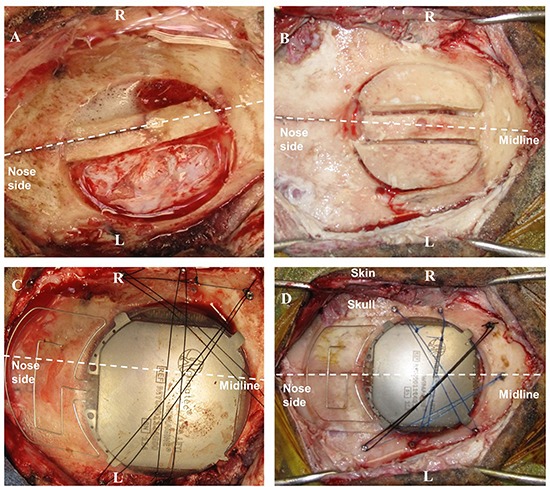
Surgery for WIMAGINE^®^ implantation in the ovine model. **(A,B)** Trepanation and division of the bone disk into 3 parts (**A:** sheep#1, **B:** sheep#2); **(C,D)** WIMAGINE**^®^** implant craniotomy fixation during surgery (**C:** sheep#1, **D:** sheep#2). (R: right side, L: left side).

Only one device could be implanted – partially covering left and right hemispheres because of the implant size (50 mm in diameter). The craniotomy to expose the epidural space was carried out in several steps. After antisepsis and draping, a linear incision was made between the nasion and the occipital bone in the midline, and the bone surface was extensively exposed. Using the midline and Bregma as an anatomical reference, we performed the craniotomy with a custom-made bone trephine (50 mm in diameter; SMAO, France) with a 1 cm shift for sheep#1 and centered for sheep#2. Because of the presence of the Superior Sagittal Sinus (SSS), the trephine was only used to cut the external table of the skull. The rest of the craniotomy was divided into three parts using a drill (Midas Rex^®^; Medtronic Inc.) so as to isolate the SSS and to avoid vascular damage (see Figures 3A,B). In sheep, the SSS has a deep groove in the internal table of the skull, presenting an additional risk of bleeding. Finally, the dura mater was adherent in several places, requiring careful removal of the last part of the disk and repairs after bone extraction.

After appropriate drilling to avoid any skull fragment between the electrodes and the dura mater, the WIMAGINE^®^ implant was inserted into the craniotomy and the antennas were aligned between the sheep orbits. The small lateral titanium wings helped to keep the implant in position, avoiding rotation of the device. As shown in Figures 3C,D, additional fixation points were provided using custom-made titanium screws and Prolene^®^ 2.0 sutures (Ethicon^®^, Johnson & Johnson, NJ, United States). Control ECoG recordings were performed throughout the surgery, both before and after skin closure. Using a sterile pouch for the base station antenna, we performed intraoperative communication tests. The implant was successfully powered and communication functions were checked. Analgesia (Buprenorphine 0.1 mg/kg) and prophylactic antibiotics (Borgal 24% trimethoprim/sulfadoxine 1 ml/15 kg) were also used postoperatively.

After hemostasis and suture of the surgical plans, a dressing (10 mm thick compress) was applied with Betadine and Tensoplast^®^ at the end of surgery. Finally, we checked the wireless communication after extubation and once the animal woke.

**FIGURE 4:**
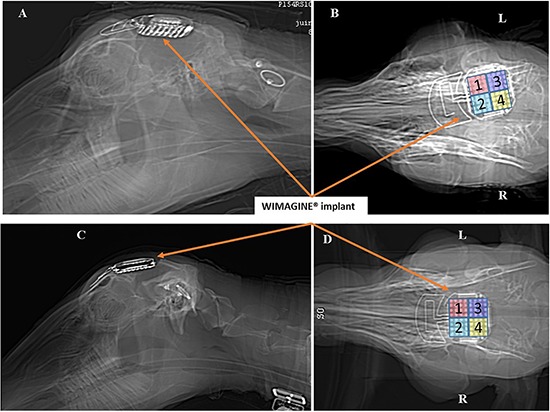
Scanner controls 3 days after surgery: Left sagittal view and right bottom view. Panels **(A,B)** for sheep #1 and Panels **(C,D)** for sheep #2. (R: right side, L: left side).

Brain scans were carried out under anesthesia 3 days after surgery (see Figure 4), 6 months later, and just before euthanasia at 10 months. There is slight angular tilt on the CT scan but it is still possible to check the position of the WIMAGINE^®^ implant in the skull. In addition, we display on this figure the locations of the four 16-contact phases.

### Experimental Design

Sessions started on the first day after surgery. Two different protocols were applied. First, once a week, we performed a session in which awake animals were trained to be calm in a sheep enclosure. In such a session, a 12-min electrocorticogram was recorded in an idle state. Second, every 2 months a SSEP session was performed under general anesthesia. We kept the anesthetic protocol chosen for the implantation since according to Baines et al. (1985), halothane which is similar to isoflurane has a limited impact on SSEP magnitude (10 to 30% decrease on peak magnitude for less than 35% of the sheep). Consequently, premedication was started with Diazepam 4.5 ml and Morphine 0.9 ml, anesthesia was induced with intravenously applied Propofol (4 mg/kg, 1/4 dose) and maintained using vaporized isoflurane (2%) in oxygen.

The tibial and median nerves of each limb were stimulated by a peripheral nerve stimulator (Energy Light©, MicroMED^®^, Italy) using two subdermal electrodes (anode proximal, cathode distal) and the SSEP were recorded using WIMAGINE^®^. For electrical stimulation, we increased the amplitude of the current until visible limb contraction was obtained between 2 and 20 mA, 100 μs pulse width and 2 Hz-train of 150–350 biphasic stimuli.

After a 10-month implantation period, the WIMAGINE^®^ implants were explanted and the brains fixed for immunohistochemical analysis.

### Signal Recording and Analysis

#### Signal Recording

Both cortical activity in an idle state and evoked potentials were recorded using the platform approved for CLINATEC’s clinical trial (Mestais et al., 2015). To ensure a robust RF communication link, we chose the 2-FSK mode of the Microsemi component (ZL70102). In this configuration the wireless data transmission is about 250 kb/s. As the Analog Digital Converter has a 12-bit resolution and the sampling frequency (SF) 976 Hz, we decided to sequentially record four 3-min long sessions for each 16-contact phases.

Chronic signal quality was quantified using raw signal power spectral density (PSD) (Bundy et al., 2014), signal-to-noise ratios (SNRs) (Nurse et al., 2018), group level signal power (*P*_*band*_) (Nurse et al., 2018), and maximum/effective bandwidth (BW) (Oxley et al., 2016; Nurse et al., 2018).

#### Raw Signal Power Spectral Density

Power spectral density of raw signal was estimated (pwelch spectral analysis, 5-s window length, 80% overlap) for each channel. The mean PSD across channels was computed for implants.

#### Group Level Signal Power *P*_*band*_

Group level signal power *P*_*band*_ was computed as the mean PSD per band for the frequency bands (0–10), (10–40), (40–100), and (100–200) Hz. The *P*_*band*_ was averaged across channels for each Phase (Phases 1–4) of each implant and is presented in 10 log_10_ scale. Frequency bands are chosen following to previous studies (Nurse et al., 2018), and as relevant frequency bands for ECoG based BCI (Wang et al., 2013).

#### Group Signal-to-Noise Ratio (*SNR*_*band*_)

Group signal-to-noise ratio (*SNR*_*band*_) was calculated as ratio of mean PSD per band *P*_*band*_ and mean PSD of noise *P*_*noise*_ in 10 log_10_ scale:

$$S\,N\,R_{b\,a\,n\,d}=10\,\log_{10}\frac{P_{b\,a\,n\,d}}{P_{n\,o\,i\,s\,e}}.$$

We estimate *P*_*noise*_ in (250–260) Hz band due to the digital filter of the Integrated Circuit (cut-off frequency 300 Hz) which start altering the signal above ∼250/260 Hz (Figure 6). The use of higher band may bias the SNRs. 10 Hz large bandwidth corresponds to Nurse et al. (2018).

Finally, the *Effective BW* was computed. Upper limit was estimated (Nurse et al., 2018) for cumulative/total noise power *CP*_noise_, calculated for band (250 – *f*_max_) Hz, *f*_max_ is a Nyquist frequency:

$$C\,P_{\text{noise}}^{+}=1.5\,\left[Q_{75}\,\left(C\,P_{\text{noise}}\right)-Q_{25}\,\left(C\,P_{\text{noise}}\right)\right]+Q_{75}\,\left(C\,P_{\text{noise}}\right),$$

where *Q*_*n*_() is *n*^th^ quantile. Then the frequency, below which *x* percent (*x* = (*P* – *CP*${}_{\text{noise}}^{+}$)/*P*) of the total power *P* of the signal are located, is computed. Effective BW is thus similar to the Spectral Edge Frequency SEFx^1^.

All statistical analyses were undertaken using GraphPad Prism (version 7.00 for Windows; GraphPad Software, La Jolla, CA, United States). In comparison to Nurse’s work for each spectrogram measured and classified in frequency bands [(0–10), (10–40), (40–100), and (100–200) Hz], a linear regression model was used to determine a linear model of the measure as a function of time (Nurse et al., 2018). The coefficients of the linear fit (intercept and slope) were then analyzed to determine the rate of change with time.

### Tissue Preparation and Histological Analysis of Brain Reactivity

At the end of this chronological study, histological investigations were performed *post mortem* to evaluate long-term effects of implantation. After freezing, two device areas were cut coronally using a freezing microtome (Leica Microsystem). Sections were collected and processed for Nissl staining and immunohistochemistry for glial fibrillary acidic protein (GFAP), and macrophage detection and microglial activation (CD11b). For the measurement of Dura mater thickness, a sample of Dura mater distant from the implantation area served as a control for the implant-covered Dura mater analysis. Three sections of Dura mater from the implant-covered area and three sections from control Dura mater were cut (30 μm thick) for Nissl staining. A measurement of the dura mater thicknesses (*n* = 100 per group) was performed using Cell Sens Science Imaging Software (Olympus). The results were represented as mean ± SD. For immunohistochemical analysis, sections were incubated with the following primary antibody solutions overnight at 4°C: anti-GFAP (1:500, monoclonal mouse IgG2b; BioRad Hercules, CA, United States) to identify astrocytes, and anti-CD11b (1/500, monoclonal mouse IgG2b; BioRad Hercules, CA, United States) to identify macrophage/microglia. Secondary antibodies (Molecular Probes – Alexa 488) were diluted to detect anti-GFAP and anti-CD11b antibodies. All sections were counterstained by incubation with the nuclear dye Propidium Iodide (Sigma-Aldrich). Sections treated only with secondary antibody served to determine non-specific binding. Tissue sections were mounted with Fluorsave reagent (Merck Millipore, France) and bound primary antibodies were visualized on a set of arbitrary defined slices, using a confocal microscope.

## Results

### *In vivo* Recording Evaluation

#### Idle State ECoG Recordings

**FIGURE 5:**
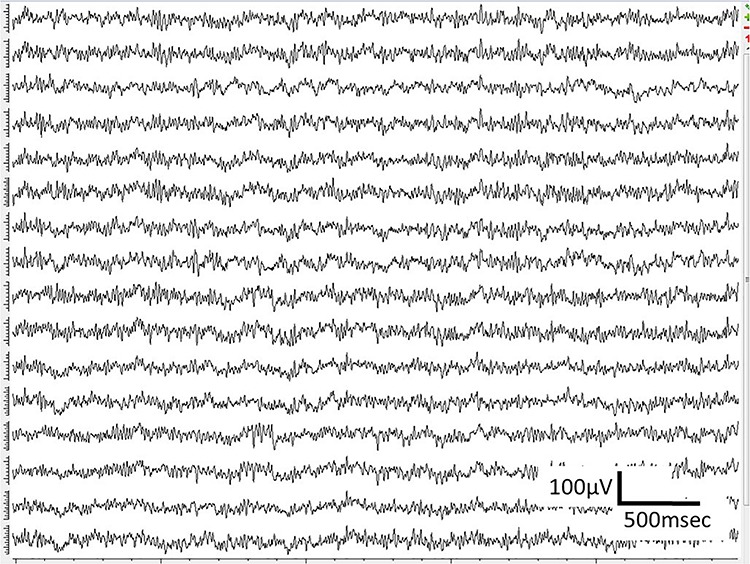
Examples of ECoG recordings in awake sheep, using the WIMAGINE^®^ platform.

**FIGURE 6:**
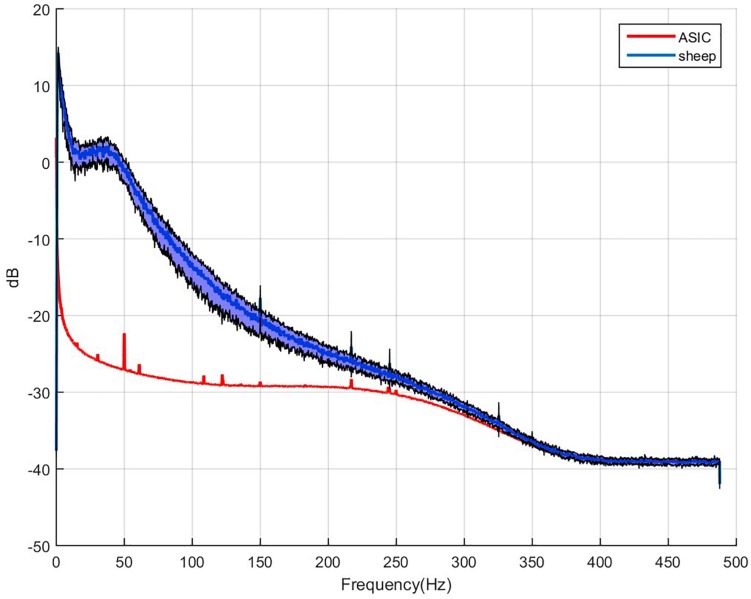
Example of 64 averaged electrode power spectral densities of ECoG during a period without chewing. *X*-axis: frequencies in Hertz, *Y*-axis: logarithm of spectral power in dB. Mean value for sheep in blue, black curves stand for the standard deviation for both animals compared to the intrinsic noise of the electronic components (labeled ASIC) with grounded electrodes.

WIMAGINE^®^ allowed us to perform wireless chronic ECoG recordings up until the end of the scheduled period of 300 days. The procedure and post-operatory events proceeded without problems. The sheep recovered immediately and were able to resume all their normal activities (walking and feeding unassisted) within a few hours. An example of the time-course ECoG is shown in Figure 5. We faced no difficulties either to connect the base station to the implant or to record from the ECoG electrodes, except for chewing artifacts (Supplementary Figure S1). Signal review demonstrated little effect of line noise in the 50 Hz band (confirmed by Figure 6) and in harmonics, so we did not apply a notch filter.

Four and a half months after implantation, sheep #2 accidentally hit one of the bars of its enclosure. The increased overall noise in ECoG recordings was a clue of implant dislodgement. From CT scans, we confirmed a significant reduction in the contact between electrode array and the dura mater. We decided to explant this WIMAGINE^®^ device, ending recording sessions with this implant at day-135 for sheep#2, which stayed until the end of the 10-month period to keep company to sheep#1 in accordance with animal husbandry best practices.

### Power Spectral Density and Signal to Noise Ratio

To estimate signal quality, we plotted the mean PSD for the two sheep, and compared these signals to the baseline, which consists of the intrinsic noise of the electronic components. Application Specific Integrated Circuit [ASIC named Cinesic and described in Robinet et al. (2011)] stands for the electronic components in Figure 6. The Nyquist frequency is 488 Hz (SF = 976 Hz) but above 250 Hz the signal spectrum is impaired by the digital band pass filter of the ASIC, and cannot be discriminate from noise.

**FIGURE 7:**
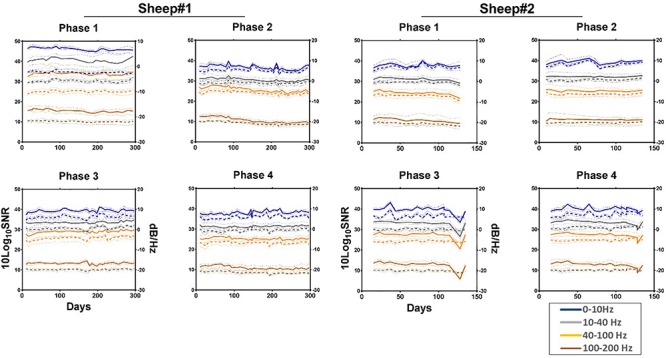
Median normalized power spectral density (solid line) and SNR (dashed line) showing their respective 95% confidence intervals (pointed lines) for each phase across four frequency bands as a function of time. All values are normalized to the size of the band. Estimated noise band: 250**–**260 Hz. Each line represents the median of the 16 channels across the given bands. Phase 1: left anterior, Phase 2: right anterior, Phase 3: left posterior, and Phase 4: right posterior area of the electrode array.

The median signal power and SNR, calculated weekly across the 16 channels for each phase of each animal are shown in Figure 7 demonstrating moderate decrease in high frequencies (Supplementary Tables S1, S2).

### Effective Bandwidth

**FIGURE 8:**
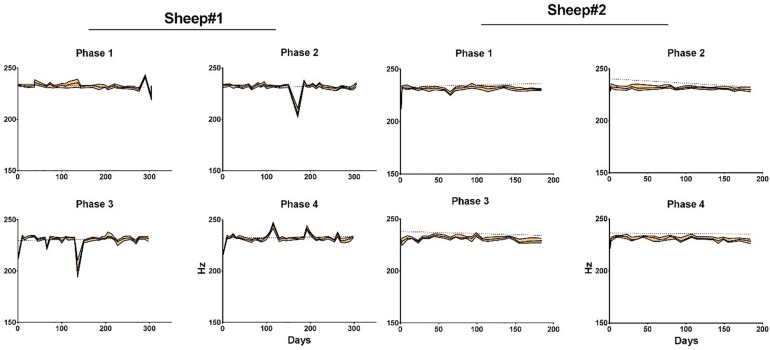
Median effective bandwidth for each phase and animal as a function of time. Areas in orange represent the 95% confidence intervals. Dotted line represents linear regression fit for each phase. Phase 1: left anterior, Phase 2: right anterior, Phase 3: left posterior, and Phase 4: right posterior area of the electrode array.

The effective bandwidth is in the range of 230 Hz for sheep #1 and #2 (Figure 8). We end the plot for sheep#2 after day 135, when the animal hit its head on one bar of the enclosure, as this dislodged the implant from the craniotomy which corresponds to a dramatic drop of the effective bandwidth. The trends of effective bandwidth for sheep #1 and #2 remained remarkably steady throughout the experiment (at least until day 135); slopes are not significantly different from zero (Supplementary Table S2).

### Somato Sensory Evoked Potentials

**FIGURE 9:**
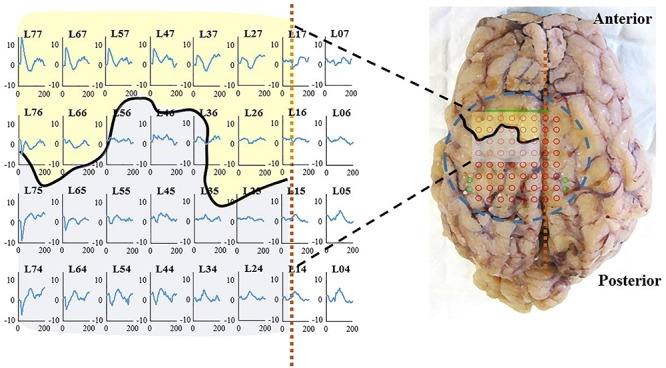
**(Left)**: SSEP at 2 months on sheep #1, right thoracic limb (right forelimb) stimulation on sheep #1: 100 μs, 2 Hz, 4 mA **(Right)**: Brain after explantation the dashed blue circle represents the contact with the inferior face of WIMAGINE^®^ with projection of the recording contacts in red and reference electrodes in green. The black curve and the dashed vertical orange line locate, respectively the central sulcus and the SSS. Primary motor cortex and somatosensory cortex are represented in transparency in yellow and gray, respectively.

Somato sensory evoked potentials are likely to arise with a latency of about 25 ms for thoracic limb stimulation according to Sanborn et al. (2011). Moreover, an inversion of the signal is expected when comparing the lower and the upper side of Rolando’s sulcus. In Figure 9 (left), a right thoracic limb activation triggered the SSEP peaks and the expected inversion in the proper cortical areas. This confirms the biological relevance of the recorded signal, in particular when studying an implant for a BCI. The location of the area recorded by the WIMAGINE^®^ implant is symbolized by a dotted rectangle (Figure 9, right). A set of three SSEP experiments with sheep#1 is presented (see Supplementary Table S3).

### Long-Term Biocompatibility Evaluation

**FIGURE 10:**
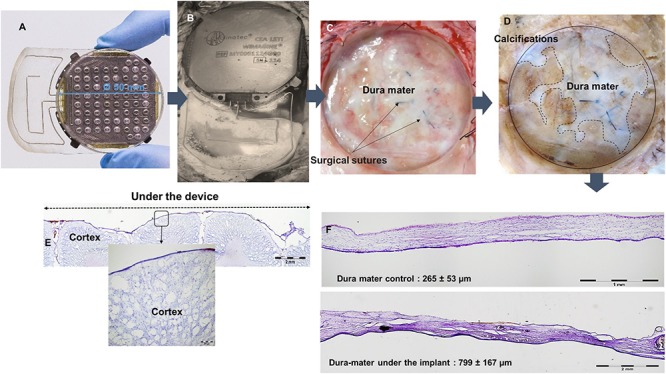
**(A)** WIMAGINE^®^ before implantation, **(B)** WIMAGINE^®^ before explantation, **(C)** aspect of the duramater after implant withdrawal, **(D)** duramater after chemical fixation. Microscopic analysis Nissl staining **(E)** control of the thickness of the duramater **(F)** cross section of the brain under the device. Data of sheep #1 after a 10-month implantation.

Histological examinations were performed post-mortem, 10 months after implantation. After euthanasia of animals and removal of skin and muscles covering the implants, observation of the implantation sites showed no encapsulation of the WIMAGINE^®^ implants (Figure 10B). Interestingly the implants were still functional after explantation. The adhesion of the electrode array and the MED-6210 over-molding silicone with tissues in contact was also tested. The WIMAGINE^®^ implants could be freely removed without damaging the dura mater. As is shown in Figure 10C, we did not observe any macroscopic sign of tissue defect but only surgical sutures used to repair adhesion spots of dura mater when the bone flap was removed. After removal of the skull, brain tissues were prepared by chemical fixation for microscopic examination in order to evaluate tissue response in contact with the WIMAGINE^®^ implant (dura mater and leptomeninges structures) and at distance (glia limitans and brain cortex). On the first part of the brain below the implant, the dura mater was removed from the brain surface and transversally cut to evaluate its thickness. As shown in Figure 10D, observations of the dura mater and the leptomeninges covered by the epidural implant revealed histopathological changes. The thickness of the implant covered dura mater was 799 ± 167 mm, whereas that of the implant-uncovered dura mater was 265 ± 53 mm (Figures 10A,E,F). As shown in Figure 10D, we observed calcification areas extending from the edges of the craniotomy to the center and these covered nearly 50% of the surface under the electrode array.

**FIGURE 11:**
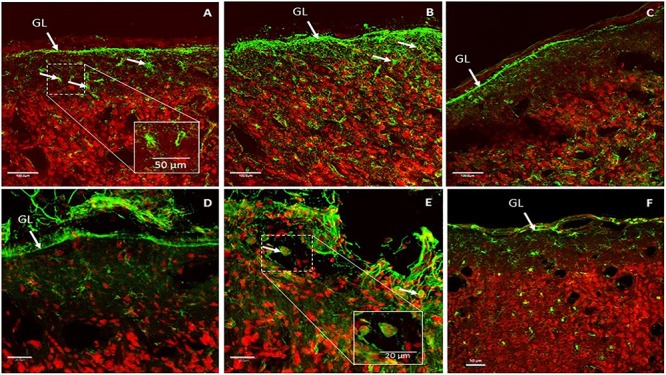
Representative glial fibrillary acidic protein (GFAP) expression patterns in implant-covered brain cortex **(A,B)** and in control brain cortex **(C)**. Scale bar = 100 μm. Representative CD11b expression patterns in implant-covered brain cortex **(D,E)** and uncovered brain cortex **(F)**. Scale bar = 20 μm **(D,E)**, 50 μm **(F)**. GL: glia limitans.

A second part of the histological studies was conducted to evaluate brain cortex reactions beneath the epidural WIMAGINE^®^ implant and to detect signs of inflammation. Reactive gliosis is the endogenous response of brain tissue to aggression and this corresponds to the accumulation and/or recruitment of glial cells (astrocytes and microglia). Glial activation is the release of glial factors that will act on target cells in the same way as the cellular immune response, and thus promote peripheral monocyte infiltration (especially macrophages and lymphocytes). Immunohistochemical studies were performed to detect signs of reactive gliosis and monocyte infiltration in the brain cortex. The results are shown in Figure 11.

GFAP is a commonly used marker to evaluate reactive gliosis as an astrocytic reaction to injury. Implant-covered brain cortex areas were characterized by elevated GFAP intensity in Glia Limitans and in layer I of the brain cortex, compared to normal cortical tissue far away from contact areas. As presented in Figures 11A,B, implant-covered brain cortex areas showed a substantial increase in GFAP reactivity, extending from Glia Limitans to layer I of the cerebral cortex. Staining revealed that astrocytes became hypertrophic; elongated with thick processes (Figures 11A,B) as compared to those in the intact brain region, which were more stellate in appearance (Figure 11C). GFAP immunoreactivity was maximum at the periphery of the contact site (Figure 11B) and declined progressively as a function of the distance from the periphery to the center of the contact area. Such elevation was observed up to 400 μm from the cortical surface.

Activated microglia and macrophages were identified via CD11b staining. The implant-covered site was characterized by a substantial increase in CD11b reactivity above Glia Limitans (Figure 11D). As is shown in Figure 11E, CD11b reactivity was increased in the peripheral contact area revealing macrophages and activated microglial cells. Some of the CD11b + cells had the appearance of large, round, blood-borne monocytes/macrophages. Smaller, process-bearing microglia were also seen at various distances from the contact site. Staining in the intact brain zone revealed resting microglia with small rod-shaped somata from which numerous thin and highly ramified processes emerged (Figure 11F).

## Discussion

The design and manufacture of cortical implants for medical use required long and expensive endeavors. Kohler et al. (2017) only identified seven teams world-wide at this level of technological readiness. Indeed, for a device to be approved for clinical trials, it is mandatory to justify the compliance of the design and manufacturing standards. In particular, Active Implantable Medical Devices directive 90/385/EEC or ISO14708-1 and ISO 13485 for quality management must be respected. Moreover, while accelerated aging tests may be interesting to determine the physical limits of the materials or the packaging hermeticity, long-term preclinical tests are mandatory to assess the implant behavior in real conditions *in vivo*. In this study, our first goal was a long-term validation of the functionality of the WIMAGINE^®^ implant and the assessment of the quality of the ECoG signal with time.

Kohler et al.’s study shares many similarities with ours. After a previous mapping experiment on sheep, this species was chosen as a model for long-term assessment of their wireless device, for which recording performances were assessed using SSEP or Auditory Evoked Potential (Gierthmuehlen et al., 2014). As far as we are concerned, we successfully recorded SSEP.

In previous preclinical studies, we published validation results of the electrode array using a semi-scale device (without electronics) on non-human primates. The signal quality and the algorithms were tested in depth using epidural electrode arrays that were wired to external WIMAGINE^®^ implants (Mestais et al., 2015). In addition, inspired by implantation for 4 months in the sheep model (Oxley et al., 2016; Torres et al., 2017) and clinical studies (Nurse et al., 2018), we extensively investigated ECoG signals over time (SNR, effective bandwidth) to validate its compliance with long-term implantation and with our final goal: enabling the “BCI and tetraplegia” clinical trial at CLINATEC (NCT02550522).

### Sheep Model Relevance for WIMAGINE^®^ Testing

In preclinical testing, implant size is important when evaluating a clinical device: a scaled-down version adapted to fit small animal models does not usually fully reflect final human models. Device size reduction could have been detrimental when extracting general conclusions prior to clinical trials. Hence, there is a need for the use of larger species when dealing with medical devices (Torres et al., 2017).

Minipigs are commonly used for preclinical studies of cuff electrodes (Bonnet et al., 2013) or brain recordings (Gierthmuehlen et al., 2011). Unfortunately, for chronic experiments in minipigs, the frontal sinus, which is an open cavity connected to the paranasal sinuses above the brain is a major technical problem for the neurosurgeon and is an obvious source of infection.

The main advantage of the sheep model is the relative compatibility of the sheep skull (biparietal distance of 80 mm) with WIMAGINE^®^ (50 mm in diameter). This set-up mimicked the implantation of the clinical device, even if only one WIMAGINE^®^ implant was placed above the sagittal sinus instead of a bilateral implantation in the clinical protocol which may limit the interest of the phases above the sinus (phase 2 for sheep#1 and phase 1 and 3 for sheep#2, see Figure 4). Moreover, the mismatch between the electrode array planar surface and the curvature of the sheep brain is more than three times larger than for human brain. Consequently the contact between the electrode array and the dura is less homogeneous. However, there is no significative difference in the effective bandwidth of the four phases.

Even though the sheep head size was adequate, cranial implantation was more arduous than expected. Indeed, the craniotomy was initiated with a 50 mm large trephine, but finished with a small drill, to smoothen the relatively rough edges of the craniotomy. Moreover, the thickness of sheep skulls ranged from 4 to 7 mm at the edges of the craniotomy, the greater thickness lying above the parietal cortex. As a consequence, the contact between the electrode array and the cortex is less homogeneous than expected for humans. Furthermore, we reported a strong adhesion of the dura to the bone flap. These difficulties are unlikely to be encountered in human surgery, because the craniotomy does not cross the midline, avoiding additional manipulations.

We oriented the implants so that the implant antennas are placed anteriorly between the eyes. In this configuration the distance between the primary (external) and secondary (implant) was less than 20 mm, whereas the wireless link was designed for a distance of between 20 and 30 mm. We never faced communication issues either in post-operative conditions or during recording sessions: wireless links sent usable neural signals during the whole protocol.

### ECoG Quality Assessment

To compare this result to the literature, we list several articles dealing with ECoG results either in preclinical or clinical long-term implantation. Using the Activa PC+S from Medtronic Inc., Swann et al. (2017) reported a decrease of the signal power for the beta and gamma band on a 1 year timeframe. Ung et al. (2017) using the NeuroVista device with subdural strip electrodes also reported a decrease in the mean values that reaches an inflection point roughly 100 days after implantation. Nurse et al. (2018) who also used subdural electrodes and the NeuroVista^®^ device reported on mean power relatively steady behavior with low variation but individual changes are far more complex, highlighting the influence of electrode location on the cortex or contact quality. Sillay et al. (2013) using RNS^®^ from NeuroPace^®^ and subdural strip reported, on a median 868-day timeframe, an impedance increase within the first 84 days followed by a stabilization of the subdural electrode impedance. Apart Schendel et al. (2014), there are very few long-term assessment of epidural signal quality in the literature. For macrocontacts (2 mm in diameter), Bundy et al. (2014) reported significantly higher PSD for epidural recordings compared to epidural between 0 and 60 Hz and the opposite above 100 Hz. But this comparison was limited to a few weeks since their patients underwent temporary placement of intracranial electrode grids to identify epileptic seizure foci.

Firstly, once implanted the WIMAGINE^®^ devices had a low sensitivity to the 50 Hz noise and its harmonics artifacts; artifacts did not increase with time. The main artifact recorded by the implant was muscular chewing activity, see Supplementary Figure S1. This is a specific characteristic of all ruminant species and was recently well-described (Oxley et al., 2016). Obviously, clinical extrapolation of the chewing artifact is not relevant.

Secondly, tissue reaction surrounding WIMAGINE^®^ did not significantly alter ECoG signals over 10 months. The ECoG signal amplitude remained relatively stable during the 10-month period (see Figure 7 and Supplementary Table S1).

The analysis of the maximum effective bandwidth showed a stable longitudinal behavior at levels of 230 Hz for both animals (Supplementary Table S2) and compare favorably with those described in the literature [level of 230 Hz vs. 100 Hz (Nurse et al., 2018); and vs. 200 Hz (Oxley et al., 2016) for epidural recording] and SNR for higher frequencies (2.6 vs. ∼1 for (40–100) Hz; 1.4 vs ∼0.2 for (100–200) Hz).

At the same time, eligibility of SNR comparison or effective bandwidth is limited by the difference in the recording device features, such as sampling rate, integrated digital filters, etc. It may be less favorable for lower sampling rate systems, or lower-pass filter (both influencing noise level estimation). Indeed, WIMAGINE device was designed for ECoG recording in the (0.5–300) Hz bandwidth. Figure 6 shows that the digital low pass filter alters the recordings above 250 Hz, 300 Hz being the cut-off frequency of this filter. Whereas Oxley et al. (2016) reported 227 and 200 ± 6 Hz, respectively for subdural and epidural contact using a Twente Medical Systems International (TMSI, Netherlands) amplifier with a low-pass filter of 553 Hz (Tinkhauser et al., 2018). Nurse et al. (2018) used the NeuroVista^®^ device with a sampling frequency of 400 Hz and band-pass filter (0.1–195) Hz and obtained an effective bandwidth in the range of 100 Hz.

### SSEP Assessment

The analysis of time evolution of epidural ECoG demonstrated a stable behavior. As the electrodes recorded information from the sensorimotor cortex (SMC) we tried to show – with anesthetized animals- phase reversal in SSEP, allowing identification of the central sulcus. We were able to identify evoked potentials on several SSEP sessions at months 2, 4 and 6 (see Supplementary Table S3). Topographical areas belonging to hind limbs and fore limbs were detected and central sulcus were also delineated at month 2 as shown in Figure 9.

### Biocompatibility Evaluation

Macroscopic post-mortem analysis confirmed the presence of tissue reactions, as previously seen in several implantable neural devices (Kozai et al., 2015). The tissue response represented here has an increase in the dura mater thickness (by a factor of 3 compared to the control), separating the recording matrix from the signal source. As discussed above, signal attenuation in this case did not alter the transmission of relevant physiological information (SSEP).

These *in vivo* trials enabled us to demonstrate the benefits of epidural implantation for an appropriate brain tissue response. The end-stage healing response to materials is usually a fibrous reaction reducing device performances. We only observed a dura mater reaction consisting of a significant thickening of this external meningeal layer. This thickening may be due to the fact that the dura mater was injured and sutured when the bone flap was removed and meningeal fibroblasts were activated. The presence of fibrotic scar tissue was confirmed by microscopic investigation. In addition to dura mater changes below the WIMAGINE^®^ implant, we observed calcifications extending from the edges of the craniotomy to the center. These calcifications could be a consequence of the craniotomy and could be explained by an osteogenic action *via* osteoforming-cells, namely osteoblasts. These cells are present in the internal periosteum and could be activated during removal of the bone flap by a regenerative process including blood factors (released upon injury of blood vessels). Due to the small curvature of sheep brain, the contact between the implant and the dura was probably too weak at the edges of the craniotomy. This could also explain the calcification.

Then we studied the effect of the WIMAGINE^®^ implant on astrocytes and microglia/macrophages *in vivo*. To study the brain cortex response below the WIMAGINE^®^ implant, we performed GFAP and CD11b labeling to detect astrogliosis and microglial activation, respectively. In this study, we observed the presence of reactive astrocytes and macrophages/activated microglia at the implantation site. In our study, GFAP + astrocytes and CD11-B + macrophages/activated microglia were observed predominantly in the periphery of the contact area and at a depth which did not exceed cortical layer 1. As a result of vascular damage, macrophages derived from the bloodstream are recruited to the injury site, and microglia – the resident immune cells of the brain- become activated.

Importantly, the effective ECoG signal bandwidth remained remarkably stable despite this observed partial calcification, combined with dura-mater thickening and superficial astrogliosis (Figure 8).

## Conclusion

This article deals with the first long-term implantation of WIMAGINE^®^, a proprietary fully implantable wireless ECoG device which compliance with the safety requirements was previously described (Mestais et al., 2015).

We highlight:

• Surgery was successfully performed on two large animals (adult sheep) and ECoG signals were recorded weekly for 10 months. For the first time, long-term SSEP and signal recordings were obtained using the WIMAGINE^®^ implant in an awake ovine model, using the acquisition platform developed for clinical trials;
• Relatively stable power spectra and SNRs of these epidural recordings. The effective bandwidth was observed at a level of 230 Hz, meaning that the high frequency bands useful for BCI decoding were preserved;
• Post-mortem analysis was carried out and showed as expected a thickening of the dura-mater below the implant but no significant inflammation or glial activation in the cortex.

The sheep animal model is different from humans in terms of anatomy and brain activity. Even if, the reported analysis of signal quality cannot be directly translated to human studies, similar methodology could be carried out on human ECoG. The outcome of this preclinical work is the first long-term *in vivo* validation of the WIMAGINE^®^ implant, confirming its ability to record brain electrical activity through the dura mater, and send digitized data wirelessly through the skin. It represents a major step toward conducting clinical trials.

## Ethics Statement

Chronic implantation experiments were conducted on two female sheep (Ovis Aries) (80–90 kg, 5 and 8 years old). All experiments were carried out following the recommendations of the European Community Council Directives of 1986 (86/609/EEC), the National Institutes of Health Guide for the Care and Use of Laboratory Animals. The Ethics Committee COMETH of Lyon, France approved the experimental protocol which was registered to the national committee under reference number 1504_V2.

## Author Contributions

FS-S, NT-M, DR, and TA wrote the manuscript. FS-S, NT-M, and CC designed the study. AL, MF, GC, and CM contributed to the experiments and provided technical support. SB, TA, TC, and A-LB contributed to the data analysis. CG and DR designed and performed the histological studies.

## Conflict of Interest Statement

The authors declare that the research was conducted in the absence of any commercial or financial relationships that could be construed as a potential conflict of interest.

## Footnotes

1 https://en.wikipedia.org/wiki/Spectral_edge_frequency
