# Supplementary material for: Long-Term Sheep Implantation of WIMAGINE®, a Wireless 64-Channel Electrocorticogram Recorder
Source: Front Neurosci. 2019 Aug 21;13:847. doi: 10.3389/fnins.2019.00847 (PMC6712079; doi:10.3389/fnins.2019.00847)
Supplement: Supplementary file 1 [file Data_Sheet_1.docx]

# Supplementary data

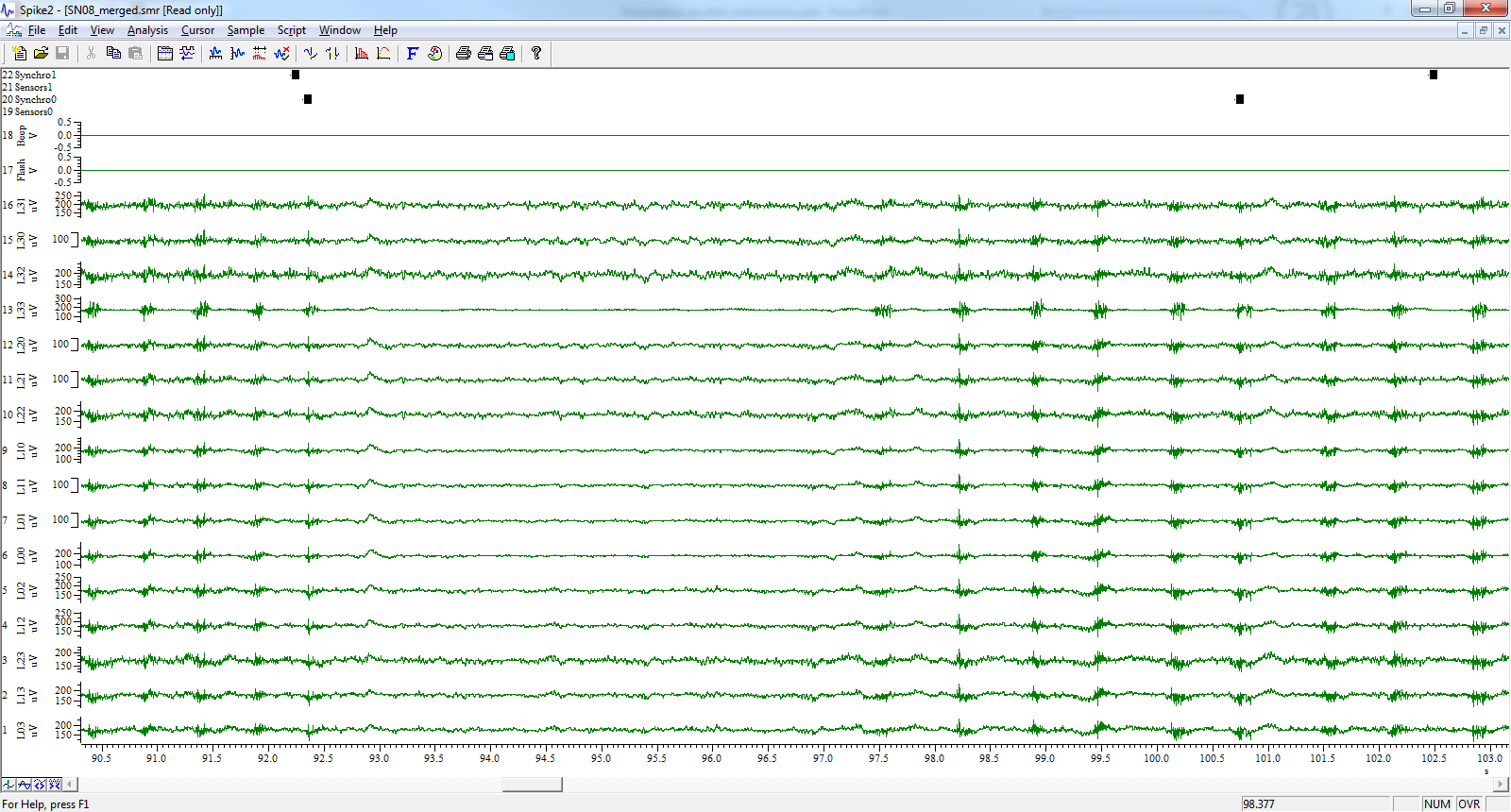


Chewing artifacts

Supplementary figure 1: ECoG monitoring on awake sheep, showing a period with chewing artifacts

|  | **Slopes of Power spectral density** | | | |
| --- | --- | --- | --- | --- |
| Frequency band | **[0-10]Hz** | **[10-40]Hz** | **[40-100]Hz** | **[100-200]Hz** |
| **Sheep #1 phase 1** |  |  |  |  |
| **Best-fit ± SE** | **-0,005 ± 0,001** | -0,0006 ± 0,001 | -0,002 ± 0,001 | **-0,006 ± 0,0009** |
| **95% Conf. Int.** | **[-0,007, -0,003]** | [-0,003, 0,002] | [-0,005, 0,0004] | **[-0,008, -0,004]** |
| **P value** | 0,0001 | 0,676 | 0,0936 | <0,0001 |
| **Sheep #1 phase 2** |  |  |  |  |
| **Best-fit ± SE** | **-0,007 ± 0,002** | **-0,007 ± 0,001** | **-0,011 ± 0,001** | **-0,013 ± 0,001** |
| **95% Conf. Int.** | **[-0,011, -0,003]** | **[-0,009, -0,005]** | **[-0,013, -0,008]** | **[-0,015, -0,011]** |
| **P value** | 0,0009 | <0,0001 | <0,0001 | <0,0001 |
| **Sheep #1 phase 3** |  |  |  |  |
| **Best-fit ± SE** | 0,003 ± 0,002 | 0,003 ± 0,0008 | 0,005 ± 0,001 | 0,0001 ± 0,001 |
| **95% Conf. Int.** | [0,0001, 0,007] | [0,002, 0,005] | [0,003, 0,007] | [-0,002, 0,003] |
| **P value** | 0,0417 | 0,0003 | <0,0001 | 0,9256 |
| **Sheep #1 phase 4** |  |  |  |  |
| **Best-fit ± SE** | 0,006± 0,002 | 0,002 ± 0,001 | 0,0002 ± 0,001 | **-0,004 ± 0,001** |
| **95% Conf. Int.** | [0,002, 0,009] | [0,0005, 0,004] | [-0,002, 0,002] | **[-0,006, -0,001]** |
| **P value** | 0,0015 | 0,0134 | 0,8398 | 0,0023 |
| **Sheep #2 phase 1** |  |  |  |  |
| **Best-fit ± SE** | 0,0002 ± 0,008 | **-0,009 ± 0,003** | **-0,016 ± 0,004** | **-0,016 ± 0,003** |
| **95% Conf. Int.** | [-0,017, 0,017] | **[-0,017, -0,003]** | **[-0,02601, -0,007]** | **[-0,022, -0,011** |
| **P value** | 0,9756 | 0,0109 | 0,0026 | <0,0001 |
| **Sheep #2 phase 2** |  |  |  |  |
| **Best-fit ± SE** | -0,0007 ± 0,009 | **-0,004 ± 0,002** | **-0,010 ± 0,003** | **-0,010 ± 0,003** |
| **95% Conf. Int.** | [-0,020, 0,019] | **[-0,009, -0,0004]** | **[-0,017, -0,003]** | **[-0,016, -0,005]** |
| **P value** | 0,939 | 0,0354 | 0,0085 | 0,0022 |
| **Sheep #2 phase 3** |  |  |  |  |
| **Best-fit ± SE** | -0,020 ± 0,012 | -0,020 ± 0,010 | -0,016 ± 0,011 | **-0,024 ± 0,011** |
| **95% Conf. Int.** | [-0,047, 0,006] | [-0,041, 0,001] | [-0,04, 0,007] | **[-0,047, -0,001]** |
| **P value** | 0,1218 | 0,064 | 0,1645 | 0,0409 |
| **Sheep #2 phase 4** |  |  |  |  |
| **Best-fit ± SE** | 0,0007 ± 0,015 | **-0,012 ± 0,005** | **-0,013 ± 0,005** | **-0,018 ± 0,006** |
| **95% Conf. Int.** | [-0,031, 0,033] | **[-0,023, -0,0008]** | **-0,024 to -0,002** | **-0,030 to -0,006** |
| **P value** | 0,9642 | 0,0369 | 0,0283 | 0,0045 |

Supplementary table 1 : Slopes of Power spectral density. Sheep #1 and #2 recorded respectively over 300 and 135 days. In bold, significant deviation of slope from zero and negative

|  | **Slopes of normalized SNR dB** | | | | **Slope** |
| --- | --- | --- | --- | --- | --- |
| Frequency band | **[0-10]Hz** | **[10-40]Hz** | **[40-100]Hz** | **[100-200]Hz** | **Effective Bandwidth** |
| **Sheep #1 phase 1** |  |  |  |  |  |
| **Best-fit ± SE** | **-0,002 ± 0,001** | 0,002 ± 0,001 | 0,0006 ± 0,001 | **-0,003 ± 0,0005** | -0,011 ± 0,009 |
| **95% Conf. Int.** | **[-0,005, -0,0002]** | [-0,0006, 0,005] | [-0,002, 0,003] | **[-0,004, -0,002]** | -0,029 to 0,007 |
| **P value** | 0,0347 | 0,1137 | 0,6439 | <0,0001 | 0,2052 |
| **Sheep #1 phase 2** |  |  |  |  |  |
| **Best-fit ± SE** | -0,001 ± 0,002 | -0,002 ± 0,001 | **-0,005 ± 0,001** | **-0,008 ± 0,001** | -0,012 ± 0,010 |
| **95% Conf. Int.** | [-0,005, 0,00]3 | [-0,004, 0,0004] | **[-0,008, -0,003]** | **[-0,009, -0,006]** | [-0,032, 0,009] |
| **P value** | 0,5154 | 0,1139 | 0,0001 | <0,0001 | 0,2535 |
| **Sheep #1 phase 3** |  |  |  |  |  |
| **Best-fit ± SE** | 0,004 ± 0,002 | 0,003 ± 0,002 | 0,006 ± 0,001 | 0,0004 ± 0,0007 | 0,009 ± 0,014 |
| **95% Conf. Int.** | [0,0005, 0,007] | [0,0003, 0,007] | [0,003, 0,008] | [-0,001, 0,002] | [-0,020, 0,039] |
| **P value** | 0,0252 | 0,032 | 0,0003 | 0,5488 | 0,5209 |
| **Sheep #1 phase 4** |  |  |  |  |  |
| **Best-fit ± SE** | 0,006 ± 0,002 | 0,002 ± 0,001 | 0,0004 ± 0,001 | **-0,003 ± 0,0007** | 0,005 ± 0,009 |
| **95% Conf. Int.** | [0,002, 0,010] | [0,0004, 0,005] | [-0,002, 0,003] | **[-0,005, -0,002]** | [-0,014, 0,024] |
| **P value** | 0,0055 | 0,0914 | 0,7303 | <0,0001 | 0,59 |
| **Sheep #2 phase 1** |  |  |  |  |  |
| **Best-fit ± SE** | 0,006 ± 0,008 | -0,004 ± 0,005 | -0,011 ± 0,005 | **-0,011 ± 0,003** | 0,017 ± 0,03 |
| **95% Conf. Int.** | [-0,011, 0,023] | [-0,014, 0,006] | [-0,022, 0,001] | **[-0,017, -0,004]** | [-0,042, 0,076] |
| **P value** | 0,4553 | 0,3986 | 0,07 | 0,0043 | 0,5648 |
| **Sheep #2 phase 2** |  |  |  |  |  |
| **Best-fit ± SE** | 0,008 ± 0,009 | 0,004 ± 0,003 | -0,002 ± 0,003 | -0,002 ± 0,002 | -0,043 ± 0,023 |
| **95% Conf. Int.** | [-0,013, 0,028] | [-0,003, 0,010] | [-0,008, 0,004] | [-0,006, 0,002] | [-0,093, 0,006] |
| **P value** | 0,4402 | 0,2256 | 0,5179 | 0,2539 | 0,082 |
| **Sheep #2 phase 3** |  |  |  |  |  |
| **Best-fit ± SE** | -0,0005 ± 0,009 | -0,0002 ± 0,003 | 0,004 ± 0,003 | -0,004 ± 0,003 | -0,020 ± 0,020 |
| **95% Conf. Int.** | [-0,019, 0,018] | [-0,007, 0,007] | (-0,003, 0,010] | [-0,010, 0,002] | [-0,063, 0,022] |
| **P value** | 0,9537 | 0,9396 | 0,3 | 0,1392 | 0,3301 |
| **Sheep #2 phase 4** |  |  |  |  |  |
| **Best-fit ± SE** | 0,019 ± 0,014 | 0,007 ± 0,003 | 0,006 ± 0,003 | 0,0007 ± 0,002 | -0,004 ± 0,026 |
| **95% Conf. Int.** | [-0,010, 0,049] | [-0,0005, 0,013] | [-0,0005, 0,012] | [-0,004, 0,005] | [-0,058, 0,049] |
| **P value** | 0,1883 | 0,0653 | 0,0686 | 0,7565 | 0,8722 |

Supplementary table 2 : Slopes of Signal to noise ratio per band and effective bandwidth. Sheep #1 and #2 recorded respectively over 300 and 135 days. In bold, significant deviation of slope from zero and negative


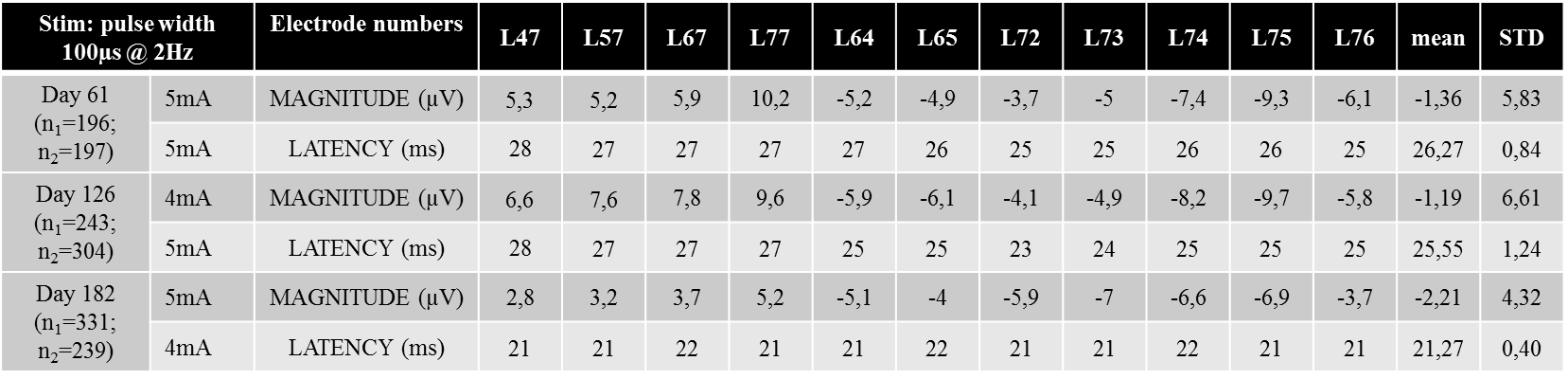


Supplementary table 3 : SomatoSensori Evoked Potentials latencies and magnitudes
